# Novel Structures of Gallenene Intercalated in Epitaxial Graphene

**DOI:** 10.1002/smll.202505640

**Published:** 2025-08-21

**Authors:** Emanuele Pompei, Katarzyna Skibińska, Giulio Senesi, Ylea Vlamidis, Antonio Rossi, Stiven Forti, Camilla Coletti, Fabio Beltram, Silvia Rubini, Lucia Sorba, Stefan Heun, Stefano Veronesi

**Affiliations:** ^1^ NEST, Istituto Nanoscienze‐CNR and Scuola Normale Superiore Piazza S. Silvestro 12 56127 Pisa Italy; ^2^ Department of Physical Science Earth, and Environment University of Siena Via Roma 56 53100 Siena Italy; ^3^ Center for Nanotechnology Innovation@NEST Istituto Italiano di Tecnologia Piazza S. Silvestro 12 56127 Pisa Italy; ^4^ CNR ‐ Istituto Officina dei Materiali (IOM) S.S. 14 Km 163.5 34149 Trieste Italy; ^5^ Present address: Faculty of Non‐Ferrous Metals AGH University of Krakow al. Adama Mickiewicza 30 30‐059 Krakow Poland

**Keywords:** gallenene, gallium, graphene, MBE, moiré, STM

## Abstract

The creation of atomically thin layers of non‐exfoliable materials remains a crucial challenge, requiring the development of innovative techniques. Here, confinement epitaxy is exploited to realize 2D gallium (gallenene) via intercalation in epitaxial graphene grown on silicon carbide. Both fabrication and characterization are conducted under ultra‐high vacuum conditions, unlike previous works on intercalated gallenene, to avoid gallium oxidation. Gallium is deposited on the graphene substrate via molecular beam epitaxy, and the intercalation is achieved by thermal treatments, leading to a homogeneous intercalation on almost the entire surface of the samples. Novel superstructures, including a striped and a hexagonal moiré pattern, are discovered and investigated via STM and LEED measurements. These structures arise from the interaction of gallenene with graphene and the silicon carbide substrate. The coexistence of different gallenene phases, including b010‐gallenene and the unprecedented 2D‐Ga(III) phase, is identified. This work sheds new light on the formation of 2D gallium and identifies a new tailored procedure for fabricating different phases of confined Ga, offering a platform for investigating the exotic electronic and optical properties of gallenene.

## Graphical abstract

This paper explores the creation of 2D gallium by intercalation beneath epitaxial graphene (Gr) on silicon carbide, achieved through confinement epitaxy. The observation of novel superstructures, including a hexagonal Gr‐12 × 12 moiré and a striped Gr‐5 × 1 pattern is reported. Atomically resolved STM measurements reveal the presence of b010‐gallenene and the 2D‐Ga(III) phase. The thermal stability of these structures is investigated, and a model is proposed to explain the diverse atomic arrangements.

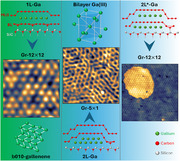

## 1 Introduction

Recently, various 2D materials have been realized through confinement at the interface between epitaxial graphene (EG) and silicon carbide (SiC) substrates.^[^ ^1^, ^2^, ^3^, ^4^, ^5^ ^]^ The confinement is achieved via the intercalation of atoms under the EG sheet. This method allows for the fabrication of atomically‐thin, ordered 2D materials over a large area. Furthermore, the graphene sheet protects the as‐produced 2D materials from the environment, making them air‐stable.^[^ ^6^ ^]^ For Group III elements, the most commonly employed technique is Confinement Heteroepitaxy (CHet).^[^ ^3^ ^]^ It involves the deposition of pure elements on the pre‐heated EG/SiC substrate in a tube furnace near atmospheric pressure, followed by thermally activated intercalation. CHet was implemented for the first time for the fabrication of 2D gallium nitride^[^ ^1^ ^]^ and successively for indium nitride.^[^ ^7^ ^]^ Besides III‐nitrides, this process can be applied to oxides^[^ ^8^ ^]^ and metals.^[^ ^3^, ^9^ ^]^ For Group III metals, this is not the only method. In particular, atomically thin gallium layers (gallenene) have been obtained by intercalation of gallium in graphene, starting from the molten metal.^[^ ^5^, ^10^ ^]^

These techniques allow for the fabrication of large‐area, high‐quality, 2D sheets of non‐exfoliable materials. Additionally, the materials are protected from environmental contamination and oxidation by the graphene capping layer. Moreover, the process is easy to scale up to the wafer size. Therefore, it opens avenues for the development of new nanoelectronic devices. Furthermore, this approach is useful for investigating exotic properties of the materials emerging at the 2D limit, such as superconductivity,^[^ ^3^, ^11^ ^]^ spin polarizability,^[^ ^12^, ^13^ ^]^ topological states,^[^ ^14^ ^]^ and nonlinear optical response.^[^ ^15^, ^16^ ^]^

Despite the significant interest dedicated to this subject in recent years, there are still open questions on the atomic structure of the confined material. Especially the atomic arrangement of ultra‐thin gallium is not well understood. Owing to the weak Ga─Ga bonds, gallium exhibits characteristics such as a melting point close to room temperature and a rich phase diagram comprising many crystalline phases. The stable phase under ambient conditions is α‐gallium.^[^ ^17^ ^]^ The most investigated forms of gallenene are a100‐gallenene^[^ ^10^, ^18^, ^19^ ^]^ and b010‐gallenene.^[^ ^10^, ^19^ ^]^ These, as suggested by their names, are derived from the (100) and (010) crystal planes of the orthorhombic lattice of bulk α‐gallium. The former is the analogue of graphene and exhibits a honeycomb lattice, while the latter is composed of a buckled hexagonal lattice. β‐gallium is a metastable phase of gallium.^[^ ^20^ ^]^ Its 2D form has been experimentally observed,^[^ ^21^ ^]^ and theory predicts that it can be achieved by relaxing a100‐gallenene.^[^ ^22^ ^]^ Furthermore, theoretical studies predict several other stable gallenene allotropes. Examples are those emerging from the γ phase of Ga^[^ ^23^ ^]^ and the variety of stochastically produced structures discussed in ref. [24]. Finally, the centered tetragonal Ga(III) is one of the most elusive phases of Ga owing to the high‐pressure required for its formation.^[^ ^25^ ^]^ Nevertheless, evidence of the formation of thin islands of Ga(III) exists.^[^ ^21^ ^]^

The scope of this work is to shed light on the atomic configuration of 2D‐Ga intercalated in EG and the emerging superstructures through surface‐sensitive characterization techniques. We have fabricated and characterized samples of 2D gallium intercalated in epitaxial graphene. Via scanning tunneling microscopy (STM) and low‐energy electron diffraction (LEED) measurements, we observed the appearance of unprecedented gallenene structures. The arrangement of gallium atoms has been analyzed to explain the emerging structures and compared to models.

## 2 Results

All 2D‐Ga samples were fabricated starting from epitaxial graphene grown on SiC (refer to Section Graphene Growth). Then, Ga is evaporated on the EG in a molecular beam epitaxy (MBE) chamber (see Section Gallium Deposition). Three samples (EG1‐EG3) were prepared with different deposition conditions. Finally, the intercalation of Ga atoms beneath the graphene sheet is achieved by thermal annealing under ultra‐high vacuum (UHV).

### 2.1 2D Gallium Intercalation

During the deposition of Ga in the MBE chamber, the pristine EG samples are maintained at a constant temperature of 500 °C. Right after Ga evaporation, intercalation is limited. From AFM measurements performed prior to annealing (see Figure S3, Supporting Information), we measured that the intercalated area is initially about 14% with respect to the total monolayer graphene area. Whereas, the majority of Ga remains on top of the EG in the form of large droplets (with an average diameter of ≈60 nm). This occurs because the deposition time scale is 5 to 10 s (refer to Table I, Supporting Information), whereas the intercalation and diffusion of Ga atoms beneath the EG requires longer time to occur. Therefore, thermal annealing has been performed after the deposition to achieve intercalation.

**Figure 1:**
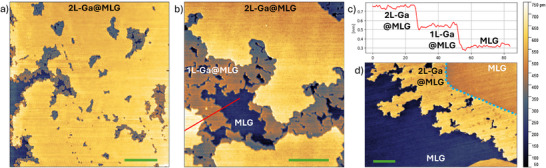
a) STM image (0.4 V, 0.8 nA) of 2D‐gallium intercalated over large area. 2L‐Ga@MLG is represented in yellow, pristine MLG in blue. b) STM image (0.1 V, 0.7 nA) of an area in which both layers of an intercalated island are visible. The upper level (2L‐Ga@MLG) is represented in yellow, the lower (1L‐Ga@MLG) in orange, and MLG in blue. c) Height profile acquired along the red line in (b), which shows that both steps are about 0.2 nm high. d) STM image (1.0 V, 1.0 nA) of an intercalated area originating from a SiC step edge (indicated by the light blue dashed line). Scale bars in (a), (b), and (d) are 100, 50, and 50 nm, respectively. Right: color scale for (a), (b), and (d).

On sample EG1, mainly composed of monolayer graphene (MLG), about one monolayer of Ga atoms was deposited. After the first annealing step at 200 °C for 12 h under UHV, STM data shown in **Figure** **1**a revealed the presence of µm^2^‐sized islands. Above these islands, we measured the graphene lattice with atomic resolution (see Figure S4, Supporting Information). This confirms that Ga atoms have intercalated below the graphene sheet. The intercalated areas cover approximately 50% of the total area of the sample, and the intercalated islands are uniformly distributed. Figure 1b shows that the islands exhibit two distinct height levels. The step height between these levels was measured via STM and is shown in the height profile in Figure 1c. The step height between MLG and the first level is 0.201 ± 0.011 nm, and a similar height of 0.203 ± 0.010 nm is measured between the first and second level. A height of about 0.2 nm is compatible with the height of a single layer of Ga atoms.^[^ ^26^ ^]^ Therefore, we attribute the first level to intercalated monolayer gallium (1L‐Ga@MLG), and the second level to the intercalation of two layers of Ga (2L‐Ga@MLG).

The majority of the intercalated areas is composed of 2L‐Ga@MLG. Small patches of 1L‐Ga@MLG are visible at the edges of the 2L‐Ga@MLG areas. Rarely, we observed regions of three‐layer intercalated Ga (3L‐Ga@MLG) (cf. Figure S5, Supporting Information). The motion of the STM tip during the scans displaces Ga atoms within the 3L‐Ga@MLG, continuously altering the morphology of the islands (as shown in Figure S5, Supporting Information). However, the graphene overlayer remains intact (the formation of defects in the graphene sheet is not observed) and rearranges to follow the motion of the underlying Ga atoms and adhere to them. A previous study observed that the third layer exhibits a different atomic arrangement with longer bond lengths.^[^ ^3^ ^]^ This is consistent with our observation that 3L‐Ga@MLG islands appear to be unstable, differently from the first two layers.

Figure 1d shows an intercalated area originating at a SiC step edge (highlighted by a light blue dashed line). This suggests that discontinuities in the graphene sheet, such as step edges of the substrate, promote the intercalation of Ga. However, there is no evidence for a preferential path for the intercalation toward the lower or the upper terrace. As Ga atoms intercalate in graphene, they diffuse for hundreds of nanometers, creating the intercalated islands. Our observation is consistent with previous results on intercalated graphene.^[^ ^27^ ^]^ Besides substrate step edges, defects within the graphene are also known to be major sites for intercalation.^[^ ^28^ ^]^ Despite the high quality of the EG samples utilized here, defects are present (as evidenced by the presence of a D peak in the Raman spectra in Figure S1, Supporting Information).^[^ ^29^ ^]^ They play a fundamental role in the intercalation of Ga at locations far from SiC step edges. Indeed, as shown in Figure 1a, large intercalated islands are observed even several micrometers away from the step edges.

### 2.2 Intercalated Gallenene Structures

**Figure 2:**
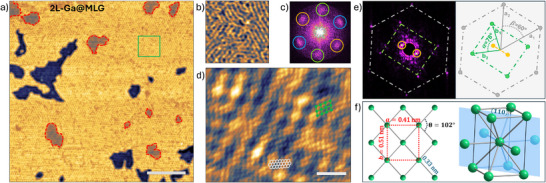
a) STM image (3.0 V, 0.8 nA) of 2L‐Ga@MLG in which the striped pattern is imaged. 2L*‐Ga@MLG regions are highlighted by the red dashed lines. b) Close‐up of (a) (corresponding to the green square in (a)) for a better visualization of the striped pattern. c) FFT of (a). The observed hexagonal pattern is given by three pairs of spots, one per each orientation of the stripes (each orientation is highlighted by a different color). d) Atomically resolved STM image (0.24 V, 0.6 nA) in which the stripes, the graphene lattice, and the centered rectangular lattice of Ga(III) are visible. Sketches of the graphene and Ga lattices are superimposed on the image in white and green, respectively. e) Left: FFT of (d). Graphene spots are highlighted by the gray hexagon and spots from gallium by the green rectangle. The spots from the striped moiré (only one direction is visible here) are circled in orange. Right: scheme of the spots in which unit vectors of Ga (b_1_, b_2_) and graphene (a_1_, a_2_) are indicated as well as the angle between them. f) Left: schematic representation of the observed centered rectangular lattice of Ga(III). Right: unit cell of bulk Ga(III) where the (110) plane is highlighted in blue. Scale bars in (a) and (d) are 20 and 2 nm, respectively.

Upon closer examination of 2L‐Ga@MLG, we observe the presence of striped domains, as shown in **Figure** **2**a. For a better visualization of the stripes, Figure 2b shows a close‐up of Figure 2a. The stripes are evenly spaced by (1.2 ± 0.1) nm. They are not randomly oriented but follow a threefold rotational symmetry. The fast Fourier transform (FFT) of Figure 2a, shown in Figure 2c, captures the symmetry of the structure. The hexagonal pattern is composed of the superposition of three pairs of spots, with one pair for each rotational stripe domain.

Figure 2d shows an atomically resolved STM image of a striped domain. Here, the stripes are aligned along just one of the three possible orientations. The FFT of Figure 2d, shown in Figure 2e, reveals only two spots (highlighted by orange circles), which correspond to the orientation of this striped phase. The atomically resolved STM image (Figure 2d) and its FFT (Figure 2e) reveal that the stripes have the same orientation as the graphene lattice and correspond to a 5 × 1 structure with respect to graphene.

Interestingly, in addition to the striped phase and the graphene lattice (sketched in gray in Figure 2d), another periodicity is observed. In fact, the FFT reveals a rectangular pattern (highlighted in green). The rectangle is formed by the first‐order spots of a centered rectangular lattice. Its base vectors (b_1_, b_2_) have equal lengths and include an angle of α = 78°. A schematic representation of the spots and unit vectors is shown in Figure 2e. In real space, the unit vectors have a length of 0.33 nm and include an angle of 102°.

The emerging rectangular lattice is attributed to the presence of gallium in the Ga(III) phase, because the Ga(III) phase exhibits a centered tetragonal lattice.^[^ ^25^ ^]^ Its (110) crystal plane (depicted in Figure 2f) consists of a centered rectangular lattice with sides *a* = 0.40 nm and *b* = 0.46 nm. These values agree well with the experimental data $\tilde{a}=0.41\pm 0.02$ nm and $\tilde{b}=0.51\pm 0.03$ nm. Moreover, the measured step height between MLG and 2L‐Ga@MLG of 0.40 ± 0.02 nm is consistent with twice the distance between (110) crystal planes in bulk Ga(III) of 0.198 nm.

Ga(III) is not expected to form under ambient conditions.^[^ ^25^ ^]^ Therefore, our observation of this phase of Ga suggests that the stabilization of Ga(III) is mediated by the interaction with SiC and graphene. Indeed, the rectangular lattice of Ga(III) is not randomly oriented. As shown in Figure 2e, its major axis is aligned with one side of the hexagonal graphene ring, and as such, three orientations of the Ga(III) phase are possible.

Moreover, the orientations of the rectangular lattice and the striped pattern are strictly related. The FFT in Figure 2e shows that the spots of the striped phase are parallel to the major axis of the rectangular pattern. This, in turn, is parallel to one of the sides of the graphene hexagon. Therefore, we conclude that the striped pattern is a moiré pattern emerging from the superposition of the honeycomb lattice of graphene and the rectangular lattice of Ga(III). The three possible orientations of the striped phase are thus determined by differently oriented Ga(III) grains.

Furthermore, the periodicity λ of the moiré pattern can be calculated as given in ref. [30]:

(1) $$\lambda =\frac{b}{\sqrt{\delta^{2}+2\left(1+\delta \right)\left(1-cos\left(\theta /2\right)\right)}}\;$$

where *b* = 0.51 nm and θ = 102° are the parameters of the underlying rectangular Ga(III) lattice (as depicted in Figure 2f), and $\delta =b/(g\sqrt{3})-1$ with *g* = 0.246 nm the lattice parameter of the graphene overlayer. The formula predicts λ = 1.26 nm, consistent with the measured value of 1.2 ± 0.1 nm.

**Figure 3:**
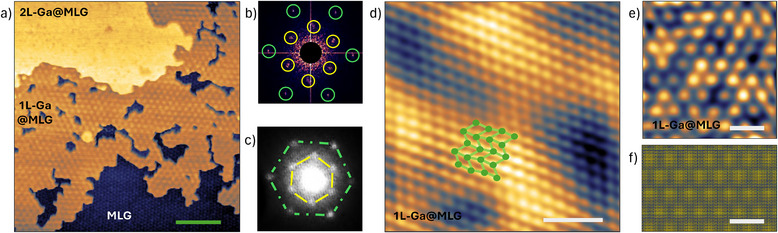
a) STM measurement (0.4 V, 0.8 nA) in a region mainly of 1L‐Ga@MLG (represented in orange). Both the SiC‐6 × 6 moiré of pristine MLG and the graphene‐12 × 12 moiré on 1L‐Ga@MLG are observed. b) FFT of (a). The SiC‐6 × 6 and the graphene‐12 × 12 spots are highlighted in green and yellow, respectively. c) LEED measurement performed on the same sample. The SiC‐6 × 6 and the graphene‐12 × 12 spots are highlighted with the same colors as in (b). d) STM image (−0.55 V, −0.31 nA) acquired on 1L‐Ga@MLG. A sketch of b010‐gallenene is superimposed onto the measured lattice. e) STM image (0.46 V, 0.12 nA) of the graphene‐12 × 12 moiré on 1L‐Ga@MLG. f) Simulated moiré created by the overlap of b010‐Ga and graphene. The scale bars in (a), (d), (e), and (f) are 20, 1, 5, and 5 nm, respectively.

Unlike 2L‐Ga@MLG, 1L‐Ga@MLG exhibits a hexagonal moiré pattern. **Figure** **3**a shows an STM image that exhibits the moiré on 1L‐Ga@MLG. This superstructure has a periodicity of 2.9 ± 0.1 nm and the same orientation as the graphene lattice. Therefore, it is a 12 × 12 modulation with respect to the graphene unit cell. The FFT of the STM image, which is shown in Figure 3b, captures the emerging 12 × 12 moiré pattern (highlighted in yellow), as well as the SiC‐6 × 6 moiré pattern of non‐intercalated EG (highlighted in green).^[^ ^31^ ^]^ A LEED measurement performed on the same sample is shown in Figure 3c. The diffraction pattern is in perfect agreement with the FFT result. This further confirms the presence of an ordered 12 × 12 moiré pattern.

Imaging 1L‐Ga@MLG with atomic resolution at a bias of +0.4 V, the graphene lattice was observed (see Figure S6, Supporting Information). Interestingly, as shown in Figure 3d, at a larger and negative bias of −0.55 V, the STM image shows a different hexagonal lattice, from which a nearest‐neighbor distance of 0.227 ± 0.05 nm is extracted. This structure is compatible with the planar projection of the buckled hexagonal lattice of b010‐gallenene.^[^ ^10^ ^]^ The observation of b010‐gallenene is in fact consistent with the appearance of the 12 × 12 moiré pattern, which emerges from the superposition of gallenene and graphene. A 12 × 12 pattern was recently reported also on Pt‐intercalated graphene.^[^ ^32^ ^]^ By comparing high‐resolution STM data of the moiré, shown in Figure 3e, and the simulated overlap between b010‐gallenene and graphene, shown in Figure 3f, the match is evident. In addition, the moiré period can be computed to be 2.93 nm (using the formula provided in ref. [33]), in good agreement with the STM measurements.

It should be noted that inside the 2L‐Ga@MLG phase there are small inclusions without the striped pattern (2L*‐Ga@MLG, indicated by red dashed lines in Figure 2a). 2L*‐Ga@MLG does not exhibit the Ga(III) rectangular lattice. Instead, it shows a hexagonal pattern analogous to the 12 × 12 moiré of 1L‐Ga@MLG (see Figure S7, Supporting Information). The surface of 2L*‐Ga@MLG appears ≈50 pm below the surface of 2L‐Ga@MLG and thus ≈150 pm above the surface of 1L‐Ga@MLG. This indicates a different arrangement of the intercalated Ga atoms, as will be discussed in Section 2.5. Despite this slight variation in the height with respect to 2L‐Ga@MLG, 2L*‐Ga@MLG is still compatible with two layers of Ga intercalated in graphene. On the contrary, given the significant height difference with 1L‐Ga@MLG, 2L*‐Ga@MLG cannot be composed of just a single layer of Ga.

In summary, we observed the coexistence of different phases of gallium in the same MLG sample, under the same conditions. This is consistent with the predicted small differences in free energy among the different phases of confined Ga.^[^ ^21^ ^]^

### 2.3 Intercalation Under the Buffer Layer

Besides the deposition of Ga on MLG, we explored the deposition of Ga on samples mainly composed of buffer layer (BL) (EG2 and EG3; see Section Gallium Deposition).

**Figure 4:**
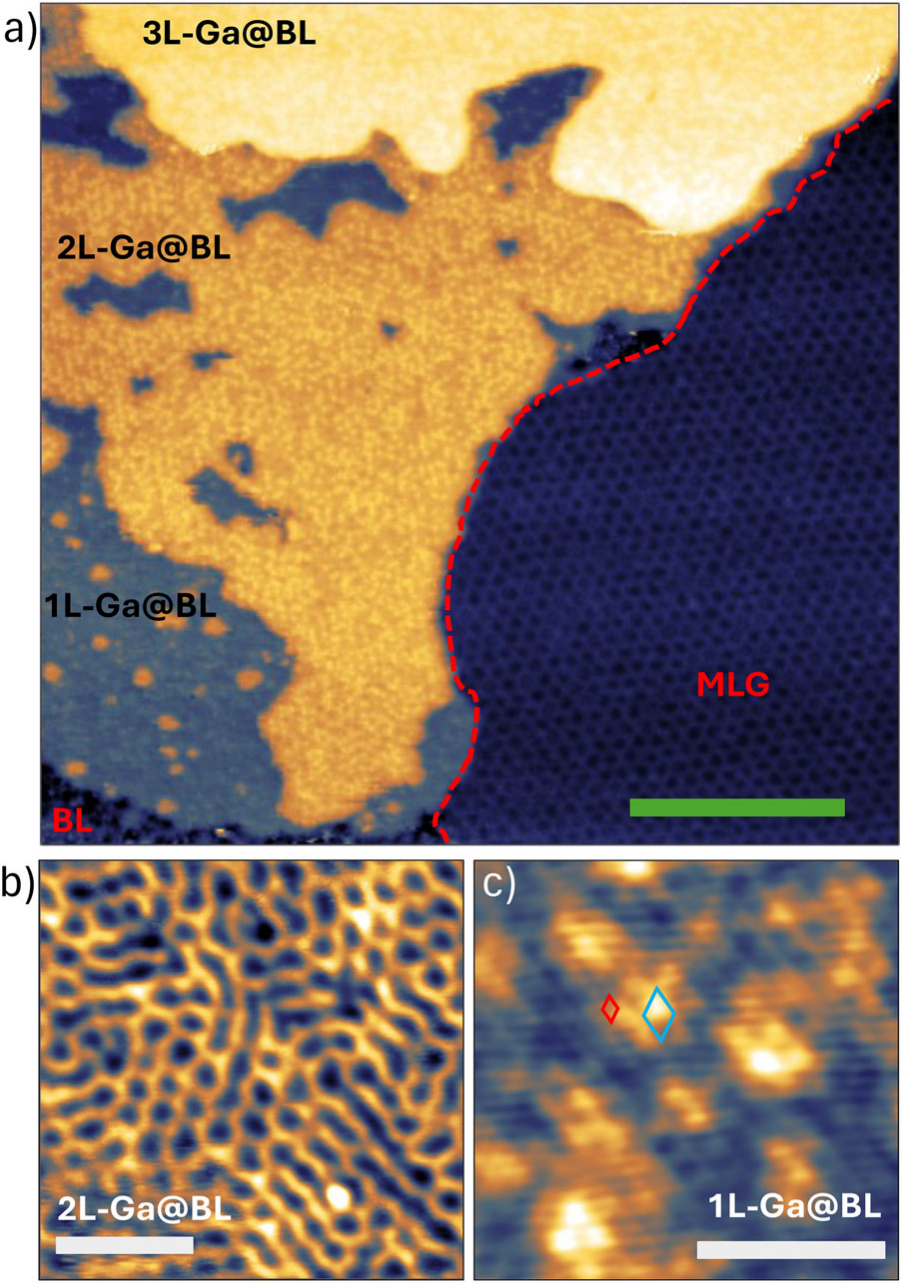
a) STM image (−2.3 V, −0.62 nA) of Ga intercalated on BL. The red dashed line indicates the edge between BL (on the left) and MLG (on the right). All three intercalated layers of Ga are imaged. b) High‐magnification STM image (2.0 V, 0.5 nA) of the striped pattern observed on 2L‐Ga@BL. c) STM image (−0.64 V, −0.24 nA) acquired on 1L‐Ga@BL exhibiting both the graphene lattice (unit cell represented in red) and the graphene‐2 × 2 reconstruction due to Ga (unit cell represented in light blue). The scale bars in (a), (b), and (c) indicate 20, 5, and 2.5 nm, respectively.

**Figure** **4**a shows an STM measurement of a BL region after deposition of Ga (acquired on sample EG3). Besides pristine BL regions that show the characteristic SiC‐6 × 6 superstructure,^[^ ^31^ ^]^ there are islands, not observed in the pristine sample, which emerge from the BL and do not show the SiC‐6 × 6 reconstruction. High‐resolution STM measurements resolved the graphene lattice above all investigated regions (see Figure S4, Supporting Information). This suggests the formation of quasi‐freestanding MLG (QFMLG)^[^ ^31^, ^34^ ^]^ due to the breaking of bonds between C atoms of the BL and Si atoms of the SiC, and the saturation of the resulting dangling bonds of the SiC substrate by Ga atoms. This, in turn, confirms Ga intercalation beneath the BL, as well.

The intercalated islands consist of three levels. The height differences between these levels extracted from STM data are 0.15 ± 0.02 nm, 0.20 ± 0.02 nm, and 0.16 ± 0.01 nm between BL and 1L‐Ga@BL, from 1L‐Ga@BL to 2L‐Ga@BL, and from 2L‐Ga@BL to 3L‐Ga@BL, respectively. Therefore, similarly to MLG, each level is given by a single layer of gallium atoms. Here, gallium mainly intercalates as a trilayer (3L‐Ga@BL). Areas of bilayer gallium (2L‐Ga@BL) are observed around the trilayer islands. Rarely, small areas of monolayer gallium (1L‐Ga@BL) are observed around 2L‐Ga@BL.

Measurements performed on 2L‐Ga@BL revealed the presence of a surface modulation resembling the striped moiré observed in 2L‐Ga@MLG. Figure 4b shows an STM measurement of the striped pattern on 2L‐Ga@BL. Here, the pattern appears less ordered compared to that in 2L‐Ga@MLG. We suggest that this is due to the higher number of defects in the BL compared to MLG. Still, both the distance between the features and their orientation are consistent with the striped moiré in 2L‐Ga@MLG. Since the striped moiré is produced by the lattices of graphene and Ga(III), as discussed in Section 2.2, this allows for the conclusion that the interaction of Ga with graphene and SiC allows for the stabilization of Ga(III) also in the intercalated BL. Moreover, the presence of the same striped superstructure in both BL and MLG samples implies that Ga(III) forms between the buffer layer and the SiC, as will be further discussed in Section 2.5.

The surface roughness of 1L‐Ga@BL and 3L‐Ga@BL (25 ± 5 pm) is substantially smaller than 2L‐Ga@BL (45 ± 5 pm). High‐resolution STM measurements on 1L‐Ga@BL and 3L‐Ga@BL reveal the presence of a 2 × 2 reconstruction with respect to graphene, as reported in Figure 4c. The 2 × 2 registry of Ga atoms results from the occupation of alternating hollow sites of the graphene lattice. This behavior has also been reported for K‐ and Rb‐intercalated graphene.^[^ ^35^, ^36^ ^]^

The intercalated areas on samples EG2 exhibit the same structures as EG3, despite the different coverage of Ga on the two samples.

The measurements performed on these samples (with 60% BL and 40% MLG) revealed that Ga is more prone to adhere to BL. AFM measurements performed right after the Ga deposition show that BL was much more decorated by Ga droplets than MLG (as shown in Figure S3, Supporting Information). Moreover, STM measurements demonstrated that Ga intercalation preferentially occurred in BL areas. This is ascribed to the higher density of defects and the larger surface roughness characteristic of the BL compared to MLG. Thus, BL offers a larger number of energetically favorable adsorption sites for gallium atoms.^[^ ^37^ ^]^

### 2.4 Thermal Stability of Gallenene Structures

Results reported in Sections 2.1, 2.2, 2.3 are related to samples annealed at 200 °C overnight under UHV. After this first thermal treatment, samples were subjected to annealing steps at gradually increasing temperatures up to 800 °C, while the evolution of the aforementioned structures was monitored.

On the MLG sample (EG1), after Ga deposition and the first annealing at 200 °C, the most common configuration is 2L‐Ga@MLG with the 5 × 1 striped moiré. Upon further annealing up to 400 °C, the 5 × 1 striped moiré is stable, whereas for temperatures higher than 400 °C, it gradually weakens, until it completely disappears at around 600 °C. In its place, a hexagonal moiré appears. This is due to the transition from 2L‐Ga@MLG to 2L*‐Ga@MLG. The emerging moiré has the same periodicity and dimensions of that observed on 1L‐Ga@MLG, that is graphene‐12 × 12. The same moiré is also observed on 3L‐Ga@MLG after annealing at 600 °C. The relaxation of all these layers to form the same moiré implies a significant increase in surface area exhibiting the 12 × 12 moiré. This is consistent with the LEED measurements performed after each annealing step. Figure S8 (Supporting Information) shows the evolution of the LEED pattern with thermal treatments. Initially, after the first annealing at 200 °C, the LEED spots of the graphene‐12 × 12 are faint, consistent with STM observations of the moiré only in small patches of 1L‐Ga@MLG and in the rare regions of 2L*‐Ga@MLG. The intensity of the 12 × 12 LEED moiré spots gradually increases with increasing annealing temperature, reaching a maximum upon annealing at 600 °C. This is in agreement with the STM observation of a gradual spreading of the hexagonal moiré of 2L*‐Ga@MLG, in place of the striped moiré of 2L‐Ga@MLG. Moreover, with successive annealing steps, the intercalated area increases from about 50% to about 90% upon annealing at 700 °C. This is due to the fact that, initially, part of the gallium remained in large droplets above the graphene sheet. Annealing facilitates the diffusion of Ga atoms from the droplets to the surrounding graphene, resulting in further intercalation. The Ga droplets are consumed during this process, but some of them are still observed on the surface of the sample even after annealing at 600 °C.

Finally, sample EG1 was heated to 800 °C. At this temperature, the de‐intercalation and desorption of Ga from the surface is observed. Figure S9 (Supporting Information) shows a comparison between optical images (acquired from the video camera of the STM chamber) of the sample at different stages of annealing. The images confirm that the largest intercalation coverage is achieved upon 700 °C annealing. After annealing at 800 °C, a large fraction of the previously intercalated areas is missing. The de‐intercalation process is known to be thermally activated in this system.^[^ ^38^ ^]^ In our work, the process occurred at much higher temperature (≈800 °C) compared to previous results (≈300 °C).^[^ ^38^ ^]^ The greater thermal stability of our samples can be motivated by the different 2D‐Ga fabrication procedures and the different defect densities of the utilized EG samples, which in ref. [38] are treated with O_2_/He plasma to increase the defectivity.

Conversely, the de‐intercalation of Ga from BL is initiated at lower temperatures, closer to the ≈300 °C reported in ref. [38]. Annealing a sample of Ga intercalated in BL at temperatures above 400 °C induces Ga diffusion to the surface. This results in the formation of Ga clusters above the BL, which are detected in STM measurements. In particular, the second and third layers of intercalated Ga are more prone to de‐intercalate, whereas 1L‐Ga@BL is still observed upon annealing at 600 °C. This is an indication of the strong interaction of Ga atoms with the SiC substrate.

Another crucial aspect of the stability of the system is its resilience against oxidation. Therefore, after the gallium intercalation, XPS measurements have been conducted on sample EG1. The acquired spectra (see Figure S10, Supporting Information) show sharp metallic Ga peaks and the absence of oxide components. This is a strong indication of the capability of graphene to protect the intercalated Ga, considering that XPS was performed after the sample was transferred in air to the XPS facility.

### 2.5 Discussion

**Figure 5:**
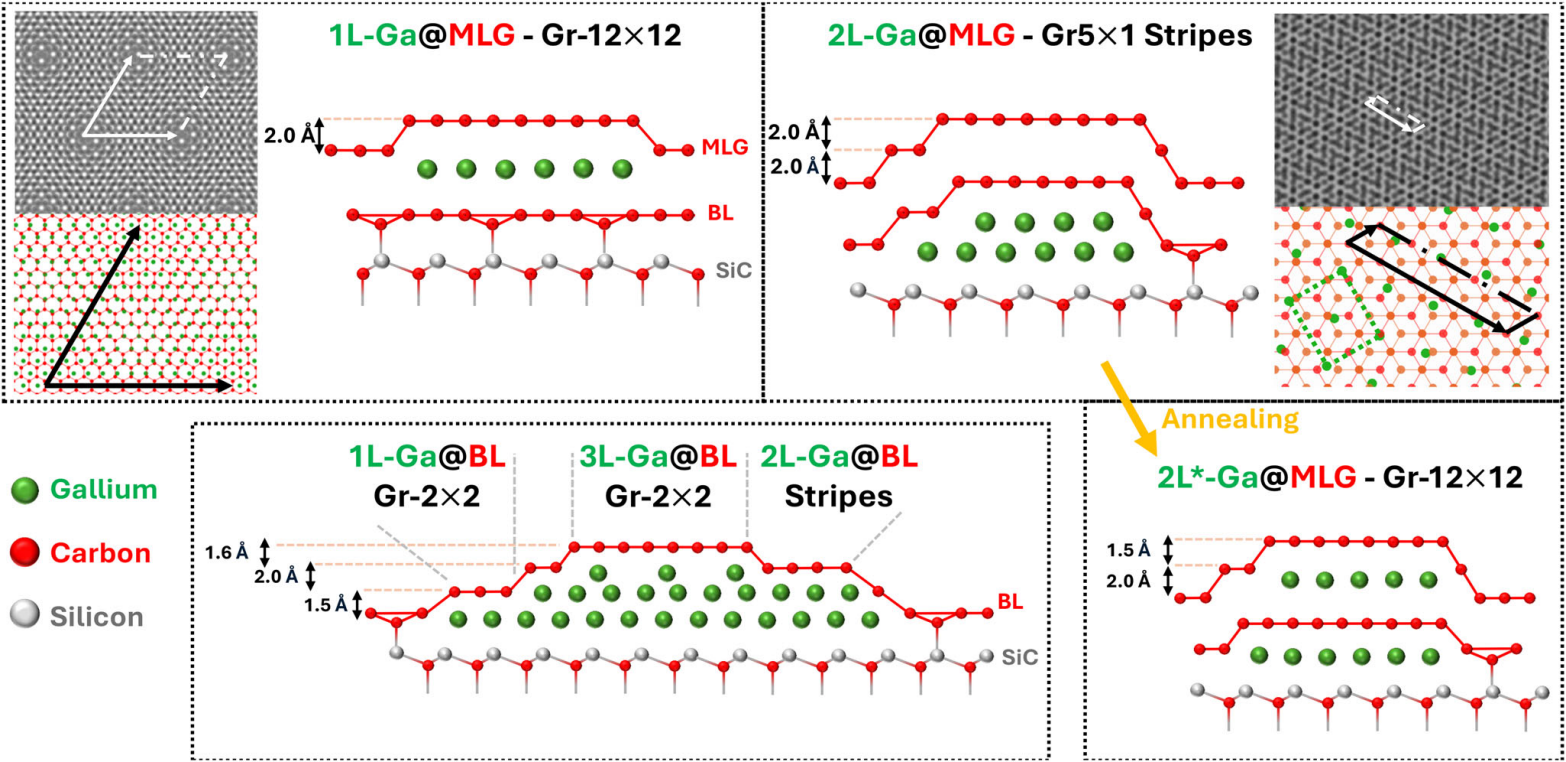
Sketch of the emerging structures of gallium intercalated in MLG and BL. Cross‐sectional views of 1L‐Ga@MLG (top left), 2L‐Ga@MLG (top right), 2L*‐Ga@MLG (bottom right), and intercalated BL (bottom left) are depicted. In addition, simulated moiré (in gray scale) and higher magnification representations of them, showing the graphene and gallium lattices, are reported both for the hexagonal Gr‐12 × 12 (top left) and the striped Gr‐5 × 1 (top right).

The evidence presented in Sections 2.2, 2.3, 2.4 allows to identify the routes for the formation of the discussed structures. In **Figure** **5** we propose a schematic representation of all structures based on our findings.

The comparison of intercalated structures on MLG and BL allows to determine the position of the gallium layers relative to the graphene sheets. Islands of Ga intercalated in BL never exhibit a hexagonal 12 × 12 moiré. This suggests that the 12 × 12 superstructure observed in 1L‐Ga@MLG in MLG corresponds to a Gr/Ga/BL/SiC configuration (see Figure 5 top left), where the 12 × 12 moiré emerges from the overlap of the topmost graphene and b010‐gallenene.

The presence of a striped pattern on both 2L‐Ga@MLG and 2L‐Ga@BL suggests that the arrangement of Ga in the striped pattern is the same in MLG and BL. This implies that Ga(III) bilayers form under the BL, where its centered rectangular lattice is stabilized by the interaction with graphene and the SiC substrate. Therefore, this results in a (2L‐)Gr/2L‐Ga/SiC configuration ('2L‐' between brackets refers to MLG; see Figure 5 top right and bottom left).

As discussed in Section 2.4, the initially small regions of 2L*‐Ga@MLG with a hexagonal moiré (cf. Figure 2a) spread and replace the striped regions with thermal annealing. The appearance of a 12 × 12 moiré occurs because of the superposition of a graphene layer onto a gallenene layer. Therefore, the transition from a striped to a hexagonal superstructure is achieved by the thermally activated climbing of part of the Ga(III) atoms above the QFMLG to rearrange into monolayer gallenene. The climbing process is consistent with the theoretical prediction in ref. [38]. Figure 5 (bottom right) shows the Gr/Ga/Gr/Ga/SiC configuration of 2L*‐Ga@MLG obtained upon climbing of Ga atoms.

Finally, this result consistently explains the fact that de‐intercalation in the BL is achieved at lower temperatures. Initially, in both 2L‐Ga@MLG and 2L‐Ga@BL, Ga atoms are located between QFMLG and the SiC substrate. If energy is provided (by annealing), Ga tends to climb above the QFMLG layer. This results in the growth of 12 × 12 moiré domains in intercalated MLG, while in BL it results in the de‐intercalation. This suggests that the initial configuration of Ga(III) is stable, but not the state of absolute minimum energy of the system. Therefore, upon annealing, the system relaxes into the most favorable configuration, which is one layer of Ga bonded to the SiC substrate and one layer above QFMLG.

## 3 Conclusion

We have provided an extensive analysis of the variety of phases and atomic arrangements which are obtained in the graphene‐Ga‐SiC system. By STM measurements we analyzed the intercalation of Ga in graphene, and the evolution of the obtained structures upon thermal treatments. Our measurements revealed the appearance of novel superstructures in this system, including a hexagonal Gr‐12 × 12 moiré and a striped Gr‐5 × 1 pattern. Atomically resolved STM measurements confirm the presence of b010‐gallenene. This appears to be the most stable form of gallenene, consistently with other experimental observations^[^ ^10^ ^]^ and theory.^[^ ^19^, ^24^ ^]^ Additionally, the rare high‐pressure phase Ga(III) was observed, and its stability was investigated. Based on our findings, we developed an interpretation of the intercalation mechanisms (presented in the schematic in Figure 5), which explains all observed atomic arrangements and superstructures. Our model is consistent with theoretical calculations on Ag‐ and Ga‐intercalated graphene.^[^ ^38^ ^]^

Unlike many of the reported works on graphene/gallenene/SiC,^[^ ^3^, ^5^ ^]^ here we deposit Ga via MBE under UHV conditions, and the intercalation is gradually induced by successive thermal treatments under UHV conditions. This allows to evolve the system step by step and to select the conditions to obtain a desired configuration. Therefore, the fabrication procedure adopted here provides a playground to investigate new properties of the system in various configurations. This can be fruitful in the investigation of the superconductivity of the Ga(III) phase,^[^ ^21^, ^24^ ^]^ in exploring the moiré physics of the system, and in understanding metal‐to‐insulator transitions in gallenene.^[^ ^39^ ^]^

The method employed here allows for the fabrication of a tunable platform in terms of atomic configuration selectivity. Additionally, the graphene capping ensures environmental stability. Moreover, this fabrication procedure is projected toward the realization of different materials, both mono‐elemental (e.g., In, Al, Sn, and Pb) and compounds (in particular, III‐V semiconductors) with potentially groundbreaking implications for the development of novel devices.

## 4 Experimental Section

### Graphene Growth

Graphene samples were epitaxially grown on the Si‐face of 6H‐SiC substrates. SiC wafers were hydrogen‐etched prior to graphenization to reduce the surface roughness. Graphene growth was achieved via thermal decomposition of SiC conducted under an Ar atmosphere in a BM‐Aixtron reactor at a temperature of approximately 1300 °C.

Three samples were produced: EG1 was composed of 85% MLG and 15% BL, while EG2 and EG3 were composed of 60% BL and 40% MLG. For more details on the pristine sample characterization, reference is made to Figures S1 and S2 (Supporting Information).

### Gallium Deposition

Pristine EG samples were loaded into a solid source Molecular Beam Epitaxy system (a Compact 21 DZ from Riber). Prior to gallium deposition, the samples were degassed in a UHV buffer chamber (base pressure 4 × 10^−9^ mbar) for 30 min at 300 °C. After degassing, the samples were moved under vacuum into the MBE growth chamber. Here, they were exposed to a flux of pure gallium atoms provided by a two‐filament Knudsen cell. During the deposition, samples were kept at a growth temperature of 500 °C (temperature measured using an IR‐pyrometer) and under rotation at 10 Hz. Different samples were subjected to different deposition conditions: growth rate and time (see Table I, Supporting Information). The growth rate was previously calibrated by Reflection High‐Energy Electron Diffraction (RHEED) Oscillations.

### Gallium Intercalation

After Ga deposition, samples were transferred in air to another UHV system. Here, they were subjected to a series of annealing steps at increasing temperatures under UHV conditions (base pressure 5 × 10^−11^ mbar). During annealing steps, samples were heated via a direct current flow through a Si substrate on top of which the samples were mounted. The temperature was monitored using an IR‐pyrometer and a thermocouple mounted on the sample holder.

### Characterization Methods

The UHV system was equipped with a LEED and an STM. STM measurements were conducted at room temperature using a VT‐UHV STM from RHK. LEED measurements were conducted using a BDL‐600IR from OCI Vacuum Microengineering (spot size ≈500 µm).

AFM measurements were performed ex situ utilizing a Dimension Icon from Bruker, operated in tapping mode.

The Raman spectrometer used was an inVia confocal Raman microscope (equipped with a 532 nm laser, with spot diameter of 1 µm) from Renishaw.

X‐ray photoemission spectroscopy was used to investigate the character (metallic versus oxide) of intercalated gallium. The sample was transferred in air to a Surface Science Instrument SSX‐100‐301 spectrometer. The spectrometer operated with an Al Kα X‐ray source and exhibits an energy resolution of 0.9 eV. The sample was degassed at 200 °C overnight before measurement, to desorb atmospheric contamination.

### Statistical Analysis

STM and AFM data were processed using the Gwyddion software.^[^ ^40^ ^]^ Raman and XPS data were analyzed using custom Python scripts.

Feature dimensions (lattice parameters, superstructure periods, and step heights) were extracted from STM data. Results were presented as mean value ± standard deviation. All analyses were conducted on a minimum sample size of 20 measurements performed at different sample locations.

## Conflict of Interest

The authors declare no conflict of interest.

## Supporting information

Supporting Information

## Data Availability

The data that support the findings of this study are available from the corresponding authors upon reasonable request.
