# Supplementary material for: Novel Structures of Gallenene Intercalated in Epitaxial Graphene
Source: Small. 2025 Aug 21;21(38):e05640. doi: 10.1002/smll.202505640 (PMC12462587; doi:10.1002/smll.202505640)
Supplement: Supplementary file 1 — Supporting Information [file SMLL-21-e05640-s001.pdf]

NANO · MICRO
small

Supporting Information

for *Small*, DOI 10.1002/smll.202505640

Novel Structures of Gallenene Intercalated in Epitaxial Graphene

Emanuele Pompei, Katarzyna Skibińska, Giulio Senesi, Ylea Vlamidis, Antonio Rossi, Stiven Forti, Camilla Coletti, Fabio Beltram, Silvia Rubini, Lucia Sorba, Stefan Heun and Stefano Veronesi**

SUPPORTING INFORMATION

Novel Structures of Gallenene Intercalated in Epitaxial Graphene

Emanuele Pompei,[1,*] Katarzyna Skibińska[‡],[1] Giulio Senesi,[1] Ylea Vlamidis,[1,2] Antonio Rossi,[3] Stiven Forti,[3] Camilla Coletti,[3] Fabio Beltram,[1] Silvia Rubini,[4] Lucia Sorba,[1] Stefan Heun,[1] and Stefano Veronesi[1,†]

[1] *NEST, Istituto Nanoscienze-CNR and Scuola Normale Superiore, Piazza S. Silvestro 12, 56127, Pisa, Italy*

[2] *Department of Physical Science, Earth, and Environment, University of Siena, Via Roma 56, 53100, Siena, Italy*

[3] *Center for Nanotechnology Innovation@NEST, Istituto Italiano di Tecnologia, Piazza S. Silvestro 12, 56127, Pisa, Italy*

[4] *CNR - Istituto Officina dei Materiali (IOM), S.S. 14 Km 163.5, 34149, Trieste, Italy*

(Dated: July 25, 2025)

[*] emanuele.pompei@sns.it

[†] stefano.veronesi@cnr.it

[‡] Currently at: Faculty of Non-Ferrous Metals, AGH University of Krakow, al. Adama Mickiewicza 30, 30-059, Krakow, Poland

I. PRISTINE EPITAXIAL GRAPHENE SAMPLES

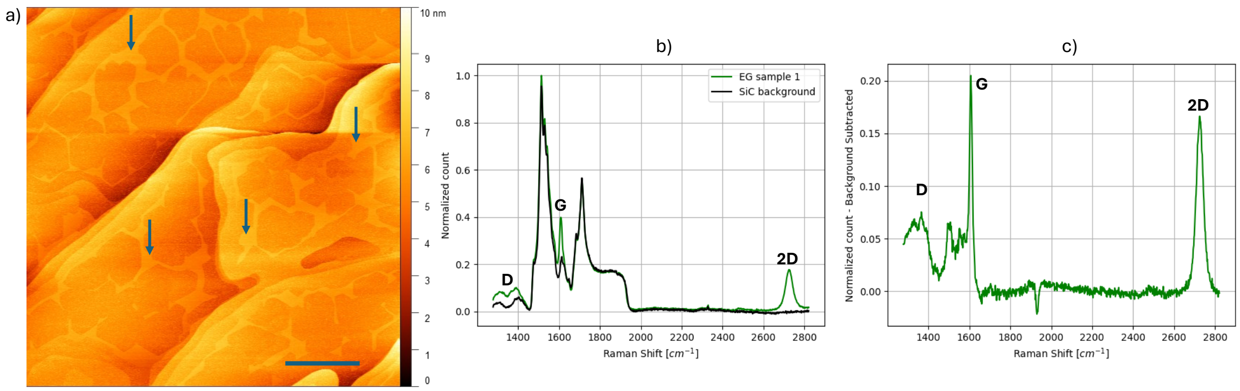

FIG. S1. (a) AFM image acquired on EG1 prior to deposition of gallium. AFM images have been utilized to estimate the MLG coverage, which is about 85%. Arrows indicate some of the BL regions. The scale bar indicates 2 μm. (b) Raman spectrum of EG1 compared to the SiC Background signal. 2D and G clearly emerge from the background as highlighted in (c), where the SiC signal is subtracted. The defect-activated D peak is observed, as well. This indicates that the graphene sheets are not defect-free [1]. The presence of defects is crucial in aiding the intercalation of gallium [2].

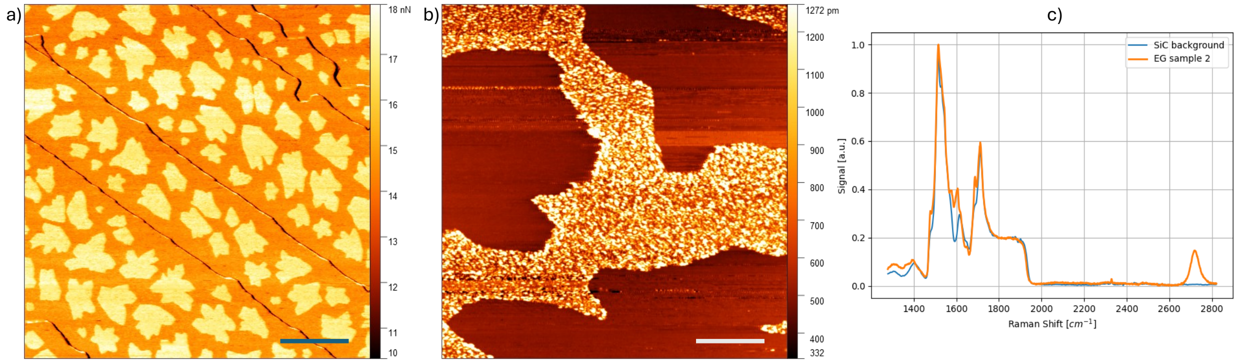

FIG. S2. (a) AFM image acquired on EG2 prior to deposition of gallium. For a higher contrast between MLG (brighter) and BL (darker) the image is presented not in height, but in adhesion scale. From AFM measurements, the MLG coverage is estimated to be about 40%. The scale bar indicates 2 μm. (b) STM image of pristine EG2 which confirms that the sample is composed of islands of MLG (flat brown areas) surrounded by BL (bright and corrugated areas). The scale bar indicates 200 nm. (c) Raman spectrum of EG2 compared to the SiC background. Despite the lower coverage of MLG, the 2D peak is evident, while the G peak is less pronounced compared to EG1.

II. GALLENENE SAMPLES

Sample	EG1	EG2	EG3
Flux [atoms/(cm^2 s)]	$\sim 6{\cdot}10^{15}$	$\sim 3{\cdot}10^{15}$	$\sim 2{\cdot}10^{15}$
Growth Rate [μm/h]	~ 1	~ 0.5	~ 0.3
Time [s]	10	5	5

TABLE I. Gallium deposition conditions for the different samples. All samples were kept at 500 °C during the Ga exposure.

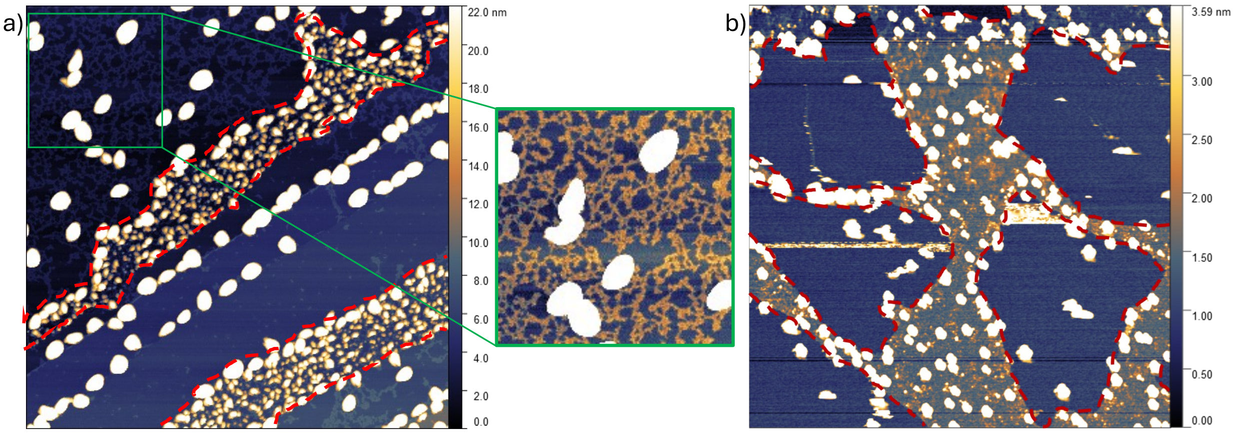

FIG. S3. 2×2 μm^2 AFM images of (a) mainly MLG sample (EG1) and (b) mainly BL sample (EG2). The boundaries between MLG and BL are highlighted by the red dashed lines. In both samples, right after Ga deposition, BL is more decorated by Ga droplets (bright spots in the images) compared to MLG. In (a), regions of intercalated gallium in MLG are observed. A close-up of one of these regions is reported. Here, the intercalated areas are orange and the MLG substrate is blue. Differently, in the mainly BL sample (b), intercalated areas in MLG are not observed, consistently with the subsequent STM measurements.

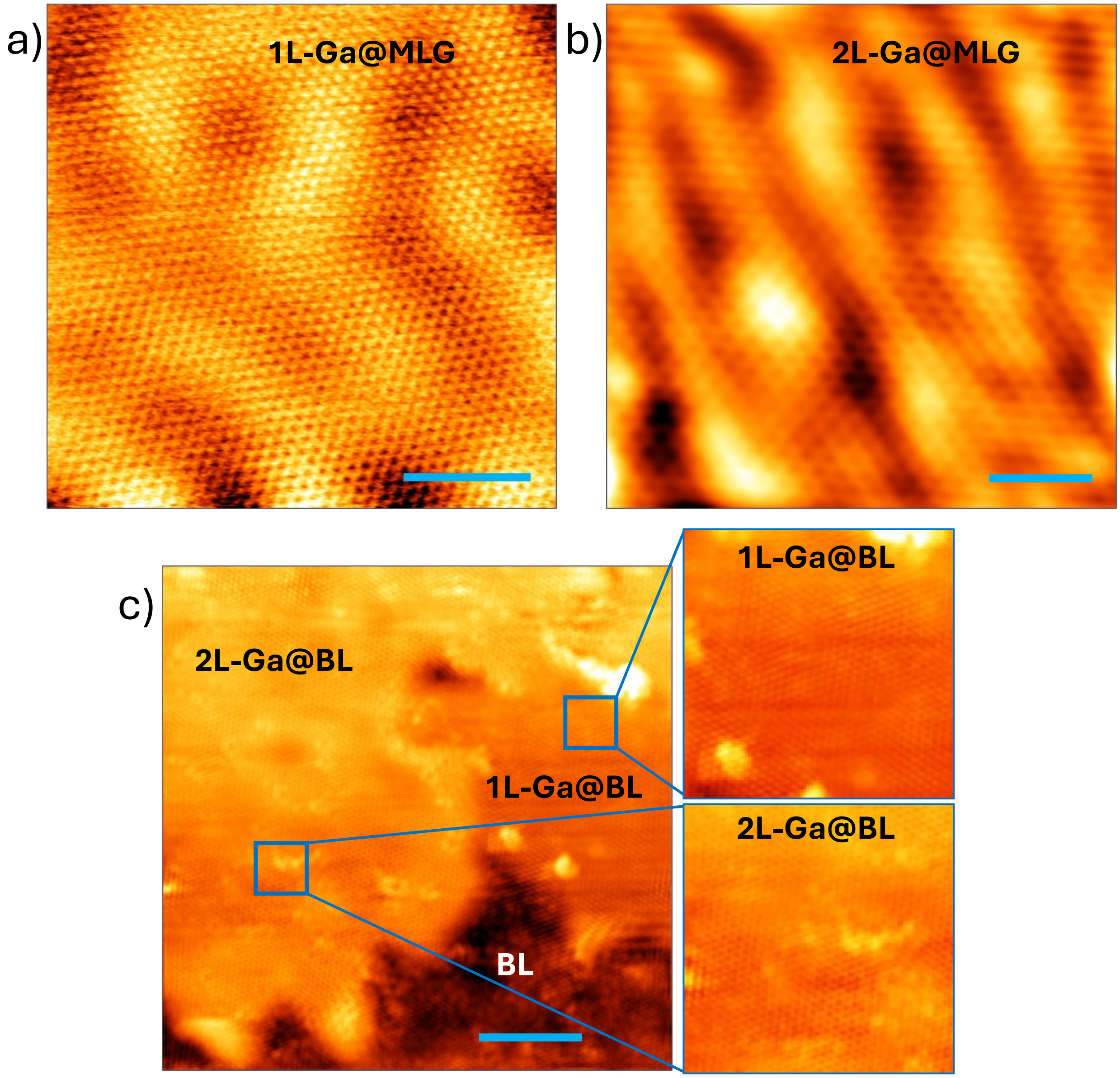

FIG. S4. Atomically resolved STM images showing the graphene lattice above (a) 1L-Ga@MLG, (b) 2L-Ga@MLG, (c) 1L- and 2L-Ga@BL. Close-ups of (c) are shown in the insets for a better visualization of the graphene lattice. The scale bars indicate 2 nm, 1 nm, and 4 nm, respectively. All images were subjected to FFT filtering.

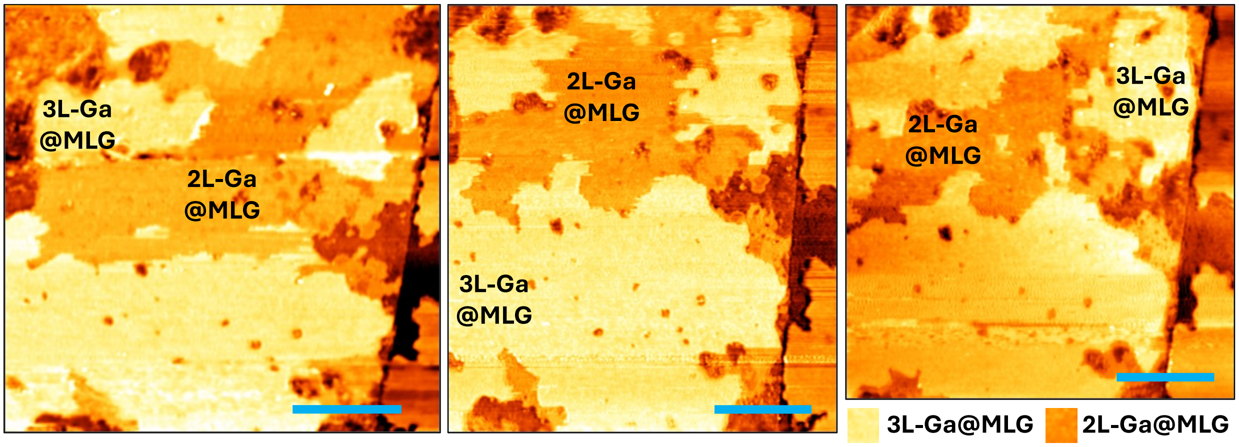

FIG. S5. STM images acquired at RT in succession on 3L-Ga@MLG on EG1 after the first annealing at 200 °C. The yellowish area, that is 3L-Ga, continuously changes shape after each scan. The scale bar indicates 50 nm in all images.

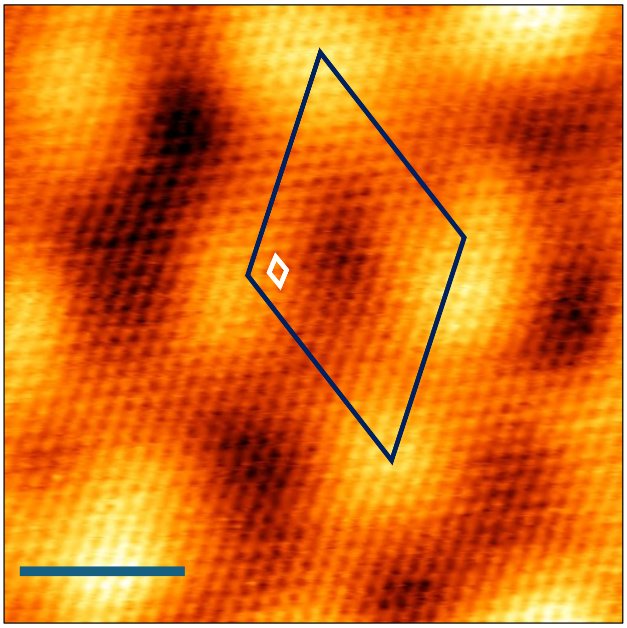

FIG. S6. STM image of 1L-Ga@MLG which shows the graphene lattice with atomic resolution. The unit cell of graphene is depicted in white and the 12×12 cell in dark blue. The image is acquired in the same region as Fig. 3(d). Here the bias applied to the tip is +0.4 V instead of −0.55 V of Fig. 3(d). The scale bar indicates 2 nm.

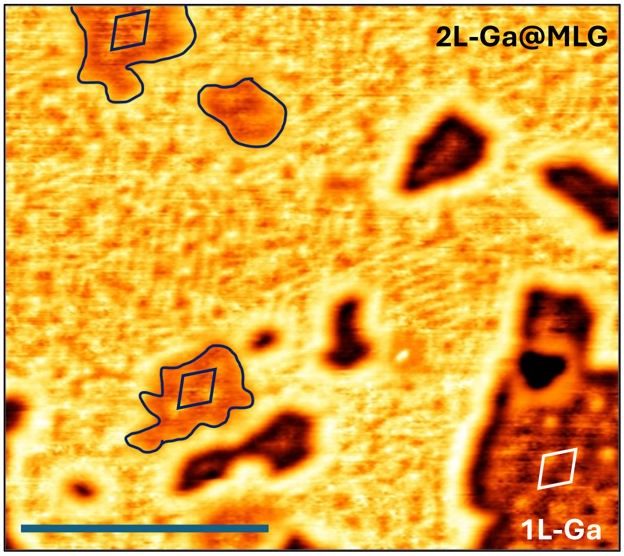

FIG. S7. STM image which shows regions of 2L*-Ga@MLG surrounded by 2L-Ga@MLG. 2L*-Ga@MLG areas are indicated by solid blue lines. These regions exhibit a hexagonal moiré which corresponds to the Gr-12 × 12 moiré observed on 1L-Ga@MLG (bottom right of the image). The unit cell of the 12 × 12 moiré is superimposed onto 2L*-Ga@MLG (blue) and 1L-Ga@MLG (white). Despite exhibiting the same moiré, 1L-Ga@MLG and 2L*-Ga@MLG are two different structures as evidenced by the clear height contrast in the STM image. The scale bar indicates 20 nm.

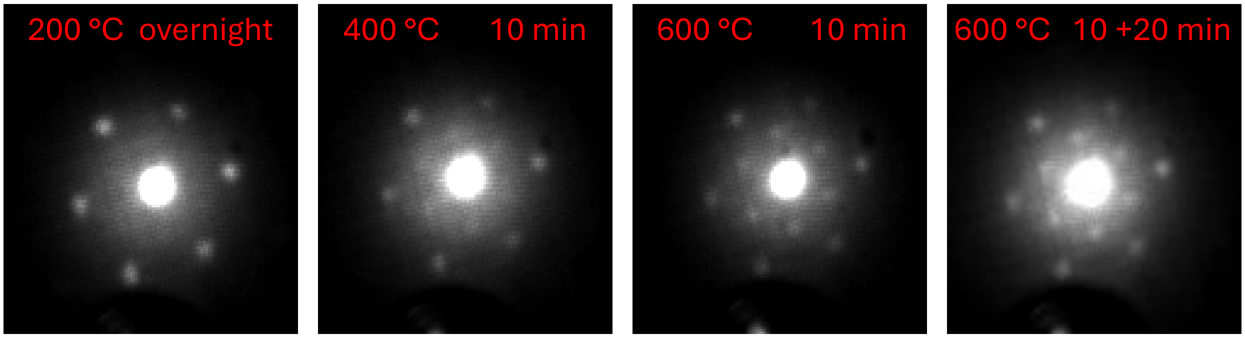

FIG. S8. Series of LEED patterns acquired on sample EG1 after every step of annealing (annealing procedure reported in each image). The larger hexagon, which corresponds to the 6×6 moiré of pristine MLG, appears to remain constant with annealing. Differently, a smaller hexagonal pattern emerges upon annealing and becomes sharper with increasing annealing temperature. It corresponds to the 12 × 12 moiré initially observed on 1L-Ga@MLG (and subsequently on 2L- and 3L-Ga@MLG).

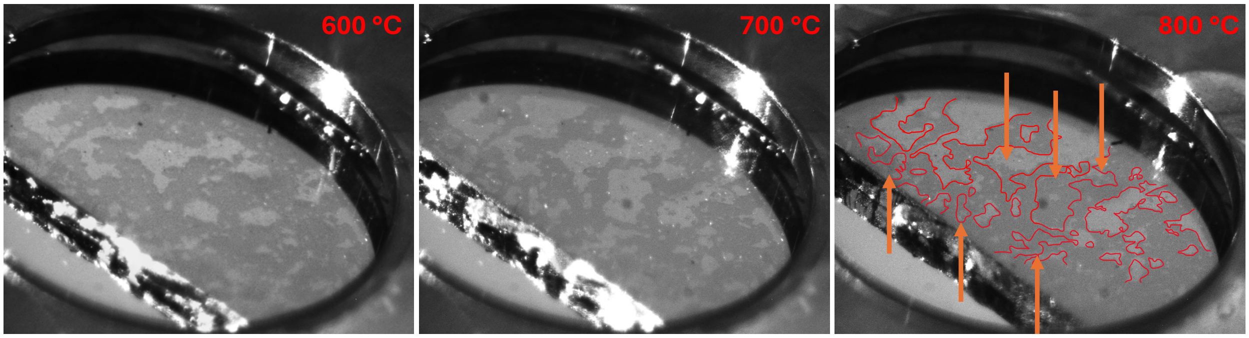

FIG. S9. Series of optical images (acquired with the video camera of the STM) of the MLG sample at different stages of annealing (annealing temperature reported in each image). From 600 °C (left) to 700 °C (center) the bright areas (regions rich in intercalated Ga) increase. Instead, upon annealing at 800 °C (right picture) the size of the bright areas is reduced. The contours of the bright areas of the central picture are superimposed onto the right picture to highlight the missing parts indicated by arrows.

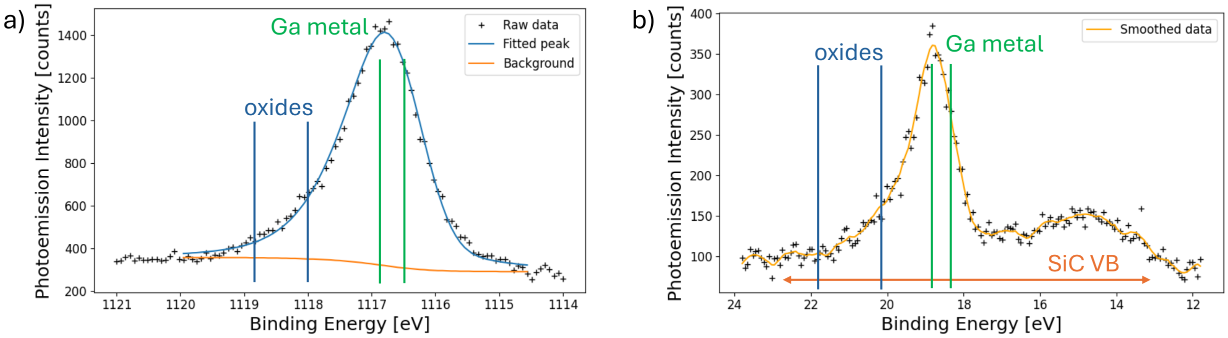

FIG. S10. (a) XPS measurement of Ga $2p_{3/2}$ emission. A single line, with the characteristic asymmetric line shape of a metal [3] is observed. Data are fitted with a Doniach–Šunjić line profile convoluted with a gaussian and referred to a Shirley-type background [4], with peak at 1116.66 eV. (b) Photoemission spectrum recorded in a wide energy region including Ga 3d emission. The weak Ga 3d peak is superimposed on a broad emission coming from valence band states of the underlying SiC [5]. Although fitting here would be difficult due to the presence of the background signal, the typical asymmetric metallic lineshape is also observed in this case, with a peak at 18.70 eV. In (a) and (b) the energy ranges reported in literature for pure metallic Ga and for Ga oxides (both native oxides and stoichiometric Ga_2O and Ga_2O_3) are marked. The same data are reported in Table II. It appears clear that the observed Ga emission arises from Ga in a metallic state.

Ref.	3d Ga metal	3d Ga_2O	3d Ga_2O_3	3d native oxide	$2p_{3/2}$ Ga metal	$2p_{3/2}$ Ga_2O	$2p_{3/2}$ Ga_2O_3	$2p_{3/2}$ native oxide
[6]	18.3	20.1	20.7		1116.4	1118	1118.7	
[7]	18.5		20.6					
[8]	18.7		20.5	20.9	1116.7		1118	1118.7
[9]	18.6		21.0	21.7	1116.5			

TABLE II. Literature data for Ga 3d and Ga $2p_{3/2}$ photoemission energy (in eV) of metallic Ga and different Ga oxides.

[1] A. C. Ferrari, J. C. Meyer, V. Scardaci, C. Casiraghi, M. Lazzeri, F. Mauri, S. Piscanec, D. Jiang, K. S. Novoselov, S. Roth, and A. K. Geim, Raman Spectrum of Graphene and Graphene Layers, Physical Review Letters **97**, 187401 (2006).

[2] N. Briggs, B. Bersch, Y. Wang, J. Jiang, R. J. Koch, N. Nayir, K. Wang, M. Kolmer, W. Ko, A. De La Fuente Duran, S. Subramanian, C. Dong, J. Shallenberger, M. Fu, Q. Zou, Y.-W. Chuang, Z. Gai, A.-P. Li, A. Bostwick, C. Jozwiak, C.-Z. Chang, E. Rotenberg, J. Zhu, A. C. T. van Duin, V. Crespi, and J. A. Robinson, Atomically thin half-van der Waals metals enabled by confinement heteroepitaxy, Nature Materials **19**, 637 (2020).

[3] S. Doniach and M. Sunjic, Many-Electron Singularity in X-Ray Photoemission and X-Ray Line Spectra from Metals, Journal of Physics C: Solid State Physics **3**, 285 (1970).

[4] D. A. Shirley, High-Resolution X-Ray Photoemission Spectrum of the Valence Bands of Gold, Physical Review B **5**, 4709 (1972).

[5] L. I. Johansson, F. Owman, P. Mårtensson, C. Persson, and U. Lindefelt, Electronic structure of 6H-SiC(0001), Physical Review B **53**, 13803 (1996).

[6] C. C. Surdu-Bob, S. O. Saied, and J. L. Sullivan, An X-ray photoelectron spectroscopy study of the oxides of GaAs, Applied Surface Science **183**, 126 (2001).

[7] R. Carli and C. L. Bianchi, XPS analysis of gallium oxides, Applied Surface Science **74**, 99 (1994).

[8] Thermofisher - Gallium X-ray photoelectron spectra, gallium electron configuration, and other elemental information, `https://www.thermofisher.com/it/en/home/materials-science/learning-center/periodic-table/other-metal/gallium.html`, 02/07/2025.

[9] XPSdatabase - Gallium, `https://xpsdatabase.net/gallium-ga-z31`, 02/07/2025.
